# Surgical management strategies for unstable sacral fractures: Case series and surgical guideline

**DOI:** 10.1016/j.ijscr.2024.110184

**Published:** 2024-08-15

**Authors:** Chonnipa Siri-archawawat, Weera Chaiyamongkol

**Affiliations:** Department of Orthopedics, Faculty of Medicine, Prince of Songkla University, Songkhla 90110, Thailand

**Keywords:** Unstable sacral fracture, Spinopelvic instability, AO type C sacral fracture

## Abstract

**Introduction:**

Unstable sacral fractures usually have posterior pelvic and spinopelvic instability, which usually be classified as AO type C. There are many controversial points concerning the management of these fractures due to their rarity. Herein, we retrospectively review cases of this injury and propose a surgical guideline.

**Case presentation:**

A 37-year-old female experienced back pain after a motor vehicle accident. Diagnostic imaging revealed an AO type C1 sacral fracture, Isler subtype 2a (case No. 7). Preoperative CT scan and CT reconstruction images showed a longitudinal fracture with a simple intraarticular fracture of the left S1 superior facet. Although the patient sustains a lumbosacral facet joint injury, the lumbosacral motion seems preservable after healing. The triangular osteosynthesis was chosen because it provides both transverse plane and vertical plane stability. The patient was allowed to continue weight-bearing ambulation after the operation. To maintain lumbosacral motion, the spinopelvic rod was removed in the postoperative seventh month.

**Discussion:**

The unstable sacral fracture might have instability in both the vertical and transverse planes. The fixation construct should provide both vertical and transverse stability. Regarding vertical fixation, it might cost a loss of lumbosacral motion, which the treating surgeon has to consider. The lumbosacral injury is sometimes trivial, and long-term lumbosacral motion is expectable. So, permanent spinopelvic fixation is not necessary. The patient's character is also an important factor. Whether the patient needs or does not need early progressive weight bearing after the operation determines the fixation method.

**Conclusion:**

Unstable sacral fractures are rare conditions usually resulting from a high-energy injury. We have proposed a surgical management strategy for this group of fractures with an optimal fixation method based on three factors: 1) fracture morphology, 2) lumbosacral integrity, and 3) concomitant injury.

## Highlights

• We presented the consecutive cases of unstable sacral fracture surgically treated in a tertiary care hospital.
• Two major biomechanical components, vertical and transverse stability, need to be considered while planning on the surgical treatment of an unstable sacral fracture.
• AO sacral fracture classification system is now the most comprehensive.

## 1 Introduction

Sacral fractures with spinopelvic instability are commonly related to high impact trauma which usually occurs in multiple trauma patients after a fall from height or motor vehicle accident [1]. The incidence of non-osteoporotic sacral fractures in the past decade was 2.09 cases per 100,000 persons and osteoporotic fractures were reported at 1–5 % of the elderly patients at risk [2,3]. The rates of associated injuries and neurological deficit are very high, which increases morbidity and mortality [4].

The sacral bone forms the posterior segment of the pelvic ring connected to the lumbar spine and functions as a keystone. Biomechanical studies have shown that axial force is transmitted through lumbosacral articulation into the upper sacrum and propagated laterally across the sacroiliac joints to the pelvis [5,6]. Many sacral fracture classifications have been developed to describe sacral fracture morphology on the basic of the degree of instability and prognosis such as the AO sacral fracture classification, which is one of the popular classifications [7,8].

The general indications for surgery of an unstable sacral fracture are the existence of instability, malalignment, and neurologic deficit. However, there are many controversial points regarding the details of the operation, such as type of fixation, need for lumbosacral arthrodesis, or hardware removal. For the unstable sacral fracture, usually referred to as AO type C and some type B, the instability can be caused by a posterior pelvic ring disruption and/or a spinopelvic junction disruption. Surgeons need to make a treatment decision based on the two major biomechanical components, the vertical and transverse axes. The goal of a fixation method is a rigid and strong construction to achieve fracture healing, while motion preservation of the lumbar spine and early mobilization in rehabilitation are also of concern. These three factors need to be prioritized individually for each patient. So, we might not need the strongest construct but the optimal fixation which is strong enough to provide the best result for the patients.

In cases of unilateral vertical sacral fracture without lumbosacral instability (AO type B), posterior stabilization or fixation in a transverse plane is usually performed due to posterior pelvic ring disruption. However, in cases of severe comminution or marked vertical displacement, a transverse plane fixation alone may not be enough [6,9]. In such cases, vertical stabilization to the lumbar spine may be needed for augmentation. In carefully selected cases, the vertical component of fixation can serve as a temporary stabilization to allow early mobilization and can be removed later to preserve lumbar motion.

A sacral fracture with spinopelvic instability (AO type C) generally needs fixation in both the horizontal and vertical planes. So, lumbopelvic fixation is commonly used due to reliable fracture reduction and stabilization in both planes and permitting early mobilization. Nevertheless, for some cases, we think that vertical stabilization can be temporarily used to preserve lumbar motion as we mentioned earlier.

Every fixation construction has both pros and cons. The most rigid construction, like lumbopelvic fixation, is not the answer for every sacral fracture. Apart from the limitation of lower lumbar motion that is the main component of lumbosacral motion, instrument-related complications including rod breakage, iliac screw prominence and wound complications have been reported at 4 to 40 %, depending on the reports [10,11]. Petryla et al. reported on patients with spinopelvic dissociation who mainly underwent posterior pelvic ring fixation alone, and showed significant decreases of functional scores at 1 year after injury compared to preinjury status [12]. In such a situation, the vertical axis stability cannot be restored by means of a transverse plane fixation or a temporary vertical plane fixation. An appropriate choice in such a case would be permanent lumbopelvic fixation.

Because of these controversial points, the rarity of cases, and the lack of well-defined guidelines for managing unstable sacral fractures, the surgical decision making is somewhat inclusive especially in the case of a sacral fracture associated with spinopelvic instability. As a result, underestimation and inadequate treatment are common, which result in poor outcomes, delayed ambulation, and chronic pain from spinopelvic instability [[10], [11], [12]].

According to the concept and what we hypothesized, we retrospectively reviewed cases of sacral fractures that were treated in our institute and explored the outcomes along with the associated complications. Then, we developed a surgical management strategy and guidelines for unstable sacral fractures.

## 2 Materials and methods

After approval by the institutional review board of the hospital, we retrospectively reviewed 20 consecutive patients diagnosed with unstable sacral fracture who were treated surgically between 2017 and 2022 in a tertiary care hospital. This work had been reported in line with the PROCESS criteria [13].

All demographic information, associated fractures, surgical data, pre- and post-operative images of the pelvis, instrument-related complications, and ambulation programs were documented. The patient identification was removed to maintain patient anonymity. Functional scores as assessed by the Oswestry Disability Index (ODI) and pain scores based on numerical rating scale (NRS) scores ranging from 0 to 10 were recorded at the final follow-ups [14].

Sacral fractures were classified by the AO classification system based on plain pelvic radiographs and computed tomography (CT) scans. An MRI was also done in cases with neurological deficit on examination. L5S1 facet joint injuries and fracture morphology were described in detail, based on Isler's classification [15,16]. The surgical modalities, which included transverse plane stabilization with/without vertical plane stabilization, were reviewed. The decision to perform lumbosacral arthrodesis depended on the attending surgeon's decision as to whether the lumbosacral joint could be preserved or not.

## 3 Results

Twenty patients were identified as unstable sacral fracture, AO types B and C, of which most were AO types C1 or C3. The mean age of the patients was 34 years, ranging from 18 to 62. The other demographic data are shown in Table 1. Fractures which had L5S1 facet joint involvement were stratified using Isler's classification and their morphologies are shown in Table 2. The common injury causes were motorcycle or motor vehicle accidents (65 %). 85 % of all cases had an associated pelvic ring injury, usually a combined type, and underwent definite fixation by orthopedic trauma surgeons in our institute.

**Table 1:** Patient's demographic data.

		*N* = 20	%
Sex	Male	9	45
	Female	11	55
Age (years)		34.2 ± 14.20	
Injury type	Motorcycle accident	11	55
	Motor vehicle accident	2	10
	Fall from height	2	10
	Gunshot	2	10
	Insufficiency fracture	1	5
	Struck-by heavy object	1	5
	Pedestrain	1	5
Associated pelvic ring injury	Yes	17	85
	Lateral compression (LC)	3	17.6
	Combined	12	70.6
	Rami	2	11.8
	No	3	15
Neurological deficit	Yes	13	65
	Motor weakness	10	50
	Numbness	9	45
	Anal involvement	4	20
	No	7	35
AO classification	B3	1	5
	C0	2	10
	C1	6	30
	C2	4	20
	C3	7	35
Isler subtype of lumbosacral joint instability	1	4	20
	2a	3	15
	2b	4	20
	2c	1	5
	3	2	10
Fixation	Lumbopelvic fixation	10	50
	Triangular fixation	6	30
	Posterior pelvic fixation alone (iliosacral screw, transiliac-transsacral screw or transiliac fixation)	4	20
Lumbosacral joint arthrodesis	Yes	10	50
	No	10	50
Decompression	Yes	6	30
	No	14	70
Complication	Broken rod	2	10
	Wound infection	1	5
	Wound dehiscence	1	5
Implant removal	Yes	3	15
	No	17	85
Follow-up time(months)		16.6 ± 11	
ODI (%) post op		8 ± 3.7	
NRS (0−10) post op		1.3 ± 1.7

**Table 2:** Chart of the patients.

No.	Sex	Age	Injury type	Associated pelvis injury	Associated musculoskeletal injuries and treatment	Neuro-logical deficit	AO classifi-cation	Isler subtype of lumbosacral joint instability	L5S1 facet joint injury/fracture morphology	Operation	Lumbosacral joint arthrodesis	Decom-pression	Mobilization before discharge	Mobilization 1st month after discharge	Complication	Implant removal	Follow-up time (months)	ODI post op (%)	NRS post op (0–10)
1	M	22	MVA	LC	Humerus and forearm fracture S/P ORIF with plate, Radial nerve injury	Motor weakness, numbness	C2	2b	Displaced intraarticular fracture	Lumbopelvic L4-iliac	Yes	Yes	Upright on bed	WB with axillary crutches	Surgical site infection need multiple debridement	No	24	8	0
2	M	28	MCA	Open pelvic fracture; LC	Tibial shaft fracture S/P nailing	Motor weakness, numbness	C2	2b	Joint subluxation	Lumbopelvic L4-iliac	Yes	No	Upright on bed	WB with axillary crutches	No	No	6	8	1
3	F	18	MCA	LC	No	Motor weakness, numbness	C3	1	Extraarticular fracture	Lumbopelvic L5-iliac	Yes	Yes	Wheelchair	WB with axillary crutches	No	No	29	8	1
4	F	22	MCA	LC + VS	Tibial plateau fracture S/P ORIF with plate	Numbness	C1	3	Comminution	Bilateral lumbopelvic + Rt SI screw	Yes	Yes	Wheelchair	WB with axillary crutches	No	No	18	6	1
5	F	62	Insufficiency fracture	Rami	No	Numbness	C0	–	–	Lumbopelvic L4-iliac	Yes,	Yes	WB with aid	WB with aid	Broken rod	No	36	8	0
6	F	30	MCA	APC + LC	Open calcaneus fracture S/P flap, Knee dislocation S/P repair ligament	Motor weakness	C3	2b	Joint subluxation	Lumbopelvic L4-iliac	Yes	Yes	Wheelchair	Wheelchair	No	No	4	NA	NA
7	F	37	MVA	APC + VS	No	No	C1	2a	Simple intraarticular fracture	Lt Triangular fixation	No	No	Wheelchair	Walking without aid	No	Yes, 7 months post op	22	0	0
8	M	22	MCA	APC + LC + VS	No	Motor weakness, numbness, anal involvement	C1	2b	Extraarticular fracture and joint subluxation	Lumbopelvic L4-iliac	Yes	No	WB with aid	WB with aid	No	No	18	8	2
9	F	34	Struck-by heavy object	LC + VS	Metatarsal fracture S/P ORIF with plate	Motor weakness	C3	1	Extraarticular fracture	Lumbopelvic L4-iliac + Transiliac transsacral screw	Yes	No	Wheelchair	WB with aid	No	No	28	10	2
10	F	23	MCA	LC + VS	Forearm fracture S/P ORIF with plate	No	C1	2c	Joint dislocation	Lumbopelvic L4-iliac	Yes	No	Wheelchair	Wheelchair	No	No	15	10	0
11	M	62	Gunshot	No	Open femur fracture S/P ORIF with plate	Anal involvement	C3	2a	Simple intraarticular fracture	Transiliac fixation + SI screw	No	Yes	Wheelchair	WB with aid	No	No	37	NA	NA
12	F	30	MCA	VS + LC	Femur fracture S/P ORIF with plate	No	B3	–	–	Lt Triangular fixation	No	No	Wheelchair	WB with aid	Broken rod	Yes, 8 months post op	10	2	0
13	F	39	Pedestrain	APC + VS	Tibial plateau fracture S/P ORIF with plate	No	C2	–	–	Bilateral SI screws	No	No	Wheelchair	WB with aid	No	No	14	6	0
14	M	18	MCA	APC + VS	Tibial shaft fracture S/P ORIF with plate	Motor weakness, numbness, anal involvement	C3	3	Comminution with joint subluxation	Lumbopelvic L4-iliac	Yes	No	Upright on bed	WB with aid	Wound dehiscence	No	30	10	0
15	F	32	MCA	LC + VS	Femur fracture S/P ORIF with plate	Motor weakness	C2	–	–	Transiliac transsacral screw + Left SI screw	No	No	Wheelchair	WB with aid	No	No	4	NA	NA
16	M	49	MCA	LC + VS	Fracture Left acetabulum, PCL injury of left knee	No	C1	1	Extraarticular fracture	Bilateral SI screws + Rt Triangular fixation	No	No	Wheelchair	Wheelchair	Lumbar pedicle screw loosening	No (deny implant removal)	6	10	5
17	M	26	Fall from height	No	Olecranon fracture S/P ORIF with plate, Calcaneus fracture on casting	No	C3	–	–	Transiliac transsacral screw + Rt triangular fixation	No	No	Wheelchair	WB with aid	No	No (deny implant removal)	3	10	5
18	M	61	Fall from height	Rami	No	No	C0	–	–	Transiliac transsacral screw	No	No	Wheelchair	WB with aid	No	No	10	12	3
19	M	28	Gunshot	No	No	Motor weakness, numbness, anal involvement	C1	2a	Simple intraarticular fracture	Transiliac transsacral screw + Rt triangular fixation	No	No	WB with aid	WB with aid	Lumbar pedicle screw loosening	No (deny implant removal)	5	4	0
20	F	40	MCA	LC + VS	Ulna fracture S/P ORIF with plate	Motor weakness, numbness	C3	1	Extraarticular fracture	Transiliac transsacral screw + Rt Triangular fixation + Transiliac fixation	No	No	Wheelchair	WB with aid	No	Yes, 12 months post op	13	16	2

APC = Anterior Posterior Compression, LC = Lateral compression, MCA = Motorcycle accident, MVA = Motor vehicle accident, ODI = Oswestry Disability Index, ORIF = Open reduction and internal fixation, SI screw = Sacroiliac screw, WB = Weight bearing, NRS score = Numerical Rating Scale score, VS = Vertical shear.

Ten patients underwent bilateral lumbopelvic fixation up to the L4 or L5 pedicle: 4 cases of AO type C3, 5 cases of AO type C1/C2 and 1 case of AO type C0. Most of these cases had iliosacral or transiliac-transsacral screw fixation for posterior stabilization in the transverse plane. All 10 lumbopelvic fixation cases underwent lumbosacral arthrodesis for long-term fusion. The cases with AO type C1 or C2 that underwent lumbopelvic fixation had advanced L5S1 facet joint injury: 3 cases of Isler type 2b, 1 case of Isler type 2c and 1 case of Isler type 3.

Triangular fixation was performed in six patients: 3 cases of AO type C1, 2 cases of AO type C3 and 1 case of AO type B3. The cases with AO type C that underwent triangular fixation had trivial L5S1 facet joint injuries: 3 cases of Isler type 1 and 2 cases of Isler type 2a. Three patients underwent hardware removal as planned to preserve lumbosacral motion. The other three patients with triangular fixation refused subsequent removal of the lumbosacral rod; two of them had lumbar pedicle loosening on the final follow-up radiography.

Four patients underwent iliosacral or transiliac-transsacral screw fixation alone: 1 case of AO type C0, 2 cases of AO type C2, and 1 case of AO type C3. All of these cases had no lumbosacral facet joint injury except for case No.11, which was AO type C3 with a simple intraarticular fracture of the L5S1 facet joint from a gunshot wound.

The mean follow-up time of the patients was 16.6 months. Postoperative complications included two patients with wound complications (infection and dehiscence) and two patients with a broken rod that was found accidentally by follow-up radiographs without a significant clinical complaint.

## 4 Discussion

The AO sacral fracture classification system is universally applicable for sacral fracture and addresses the biomechanical stability of the posterior pelvic complex and spinopelvic stability [17]. According to the original Isler's publication, the L5S1 facet joint integrity is stratified in association with a vertical sacral fracture [16]. We found H-shaped sacral fractures with the associated L5S1 facet joint injuries (Table 2). We thought that the concept of L5S1 facet joint integrity could be applied to other configurations of sacral fractures as well. We therefore integrated the concept of lumbosacral integrity into these fracture types. Following our concept, we propose a surgical strategy for unstable sacral fractures based on the AO classification system. We focus mainly on unstable sacral fractures, which include the C types, which are subclassified as C0, C1, C2, and C3, and type B with vertical instability. We also consider the Isler classification in making treatment decisions as we are concerned about lumbosacral joint integrity.

A unilateral vertical sacral fracture (AO type B) is commonly treated with posterior stabilization using an iliosacral screw, a transiliac-transsacral screw, a sacral bar, or tension band plating. However, Griffin et al. [9] found 13 % redisplacement of vertical unstable sacral fractures treated with iliosacral screw fixation. Schildhauder et al. [5,18] reported the clinical and biomechanical benefits of triangular osteosynthesis (a combination of unilateral lumbopelvic fixation and iliosacral screw) for unstable posterior pelvic fixation, which allows early weight bearing and preserves long-term lumbosacral motion. It should be noted that unilateral lumbopelvic fixation can be temporarily applied to add more stability to iliosacral or transiliac-transsacral screw fixation for highly comminuted unilateral AO type B. Sagi et al. [19] reported one-year outcomes of triangular osteosynthesis. They found a postoperative L5/S1 scoliotic deformity, and it was irreversible even after implant removal. Another point is that most patients complained of increasing low back pain and restricted motion for their activities at 4–6 months post-op. Hardware removal resulted in an improvement of lower back complaints and mobility of the lumbosacral segment. Hardware removal is controversial and is generally performed 4–6 months postoperatively. Rod breakage is one of the most common instrument-related complications, but it may not be associated with a clinical complaint. Also, hardware removal is not performed in cases of lumbosacral arthrodesis [10]. Chaiyamongkol et al. [6] proposed a transiliac fixation using two 8.5-mm iliac screws connected with a transverse rod in addition to an iliosacral screw in a vertically unstable AO type B sacral fracture. They showed superior vertical stability when adding the transiliac fixation over the iliosacral screw alone. This method of fixation is an alternative to triangular osteosynthesis but without the potential complications of lumbopelvic fixation. We suggest an iliosacral screw, a transiliac-transsacral screw, and transiliac fixation for posterior ring stabilization. Triangular osteosynthesis should be considered for highly comminuted or vertically displaced AO type B fractures.

An AO type C0 sacral fracture is a nondisplaced U-type sacral fracture that is usually seen in low-energy insufficiency fractures. This type of fracture includes bilateral longitudinal and transverse fracture lines through the upper sacrum, dissociating the spinal column from the pelvic ring, and complete disruption of the weight-bearing axis. Lumbopelvic fixation is commonly indicated for U-type sacral fractures. Despite theoretical spinopelvic instability, as these fractures are usually nondisplaced and found in an ageing population, the principle of treatment might differ from a high-energy displaced U-type sacral fracture (AO type C3). The point of concern is early ambulation due to the higher morbidity of prolonged immobilization in elderly people, but we need to weigh the risk of medical comorbidities. Nork et al. had a good clinical outcome by using bilateral percutaneous iliosacral screw insertion in a U-shaped sacral fracture. This technique was limited to non- or minimally displaced fractures due to the bony corridor for screw insertion, and patients were advised to protect weight-bearing ambulation in a brace for 2–3 months after injury [20]. Williams et al. developed a percutaneous lumbopelvic fixation technique to minimize operative time, blood loss and allow immediate mobilization without weight-bearing restrictions with good clinical results. They routinely removed hardware because spinal fusion was not performed [21]. We suggest classifying patients as either able to tolerate delayed ambulation, have multiple medical comorbidities, or are fit for surgery and need early ambulation. In cases of delayed ambulation, percutaneous bilateral iliosacral or transiliac-transsacral screw may be performed. The strong bony purchase for secure fixation might be a concern due to the higher risk of low bone density in these cases. Lumbopelvic fixation in cases of early weight bearing is needed, either by minimally invasive or open surgery.

**Fig. 1:**
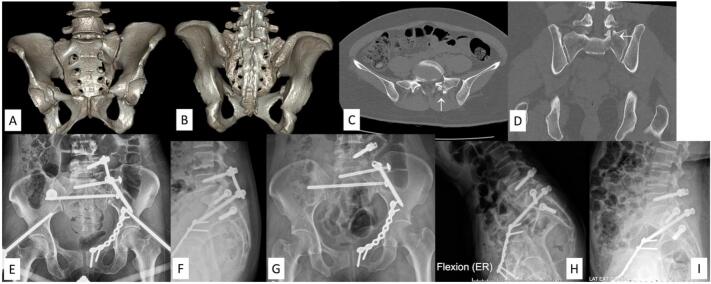
Demonstrating an AO type C1 sacral fracture, Isler subtype 2a (case No. 7). (A-D) Preoperative CT scan and CT reconstruction images showing a longitudinal fracture with a simple intraarticular fracture of the left S1 superior facet (arrow). (E, F) Postoperative radiographs after transiliac-transsacral screw fixation with left triangular fixation. (G-I) Postoperative dynamic radiographs after rod removal with preserved lower lumbar motion.

**Fig. 2:**
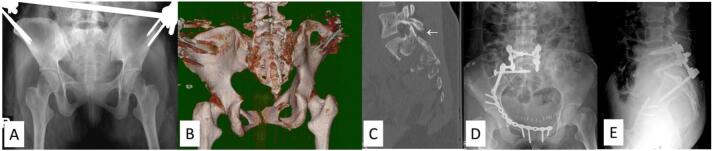
Demonstrating an AO type C1 sacral fracture, Isler subtype 3 (case No. 4). (A-C) Preoperative radiographs and CT reconstruction images showing right L5S1 joint subluxation with comminution and floating of the S1 superior facet (arrow). (D, E) Postoperative radiographs after unilateral lumbopelvic fixation, L5S1 posterior lumbar interbody fusion using a local chip graft, and a right iliac screw to reinforce the right iliosacral screw. Hardware removal was not performed in this case.

An AO type C1 sacral fracture is a unilateral vertical fracture in which the fracture line passes through or medially to the superior facet of S1. The key point of this fracture type is lumbosacral integrity. As in AO type B fractures, when there is unilateral posterior pelvic ring instability, horizontal plane fixation of the posterior pelvic ring is mandatory. Kanna et al. retrospectively reviewed cases based on Isler's subtype sacral fracture. They found that a non- or minimally displaced fracture, even if it passed medially to the L5S1 facet joint, could be treated non-operatively with a good outcome [22]. In our practice, if the lumbosacral facet joint motion can be preserved, such as in an extraarticular or simple intraarticular fracture (Isler type 1 or 2a), we perform a triangular osteosynthesis by applying an iliosacral or transiliac-transsacral screw with temporary unilateral lumbopelvic fixation without lumbosacral joint arthrodesis (Fig. 1). Otherwise, lumbosacral joint arthrodesis with lumbopelvic fixation (Fig. 2) is performed when there is L5S1 facet subluxation, dislocation, or comminution (Isler type 2b, 2c, 3).

**Fig. 3:**
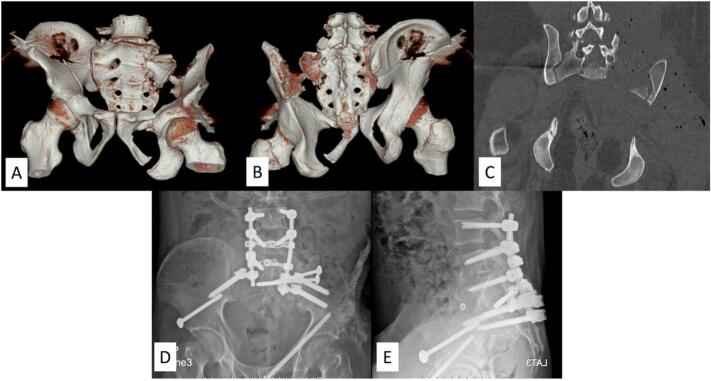
Demonstrating an AO type C2 sacral fracture, Isler subtype 2b (case No. 2). (A-C) Preoperative CT scan and CT reconstruction images showing left L5S1 facet joint subluxation and comminution of the right L5S1 facet joint. In addition, this patient had an open pelvic fracture with a following infection that required multiple debridements, resulting in severe bone loss of the left iliac wing. (D-E) Postoperative radiographs after lumbopelvic fixation to achieve secure fixation.

An AO type C2 sacral fracture is defined as a bilateral vertical fracture. Even though it has no transverse component, it also results in spinopelvic dissociation. Thus, extending vertical fixation to the lower lumbar spine needs to be considered in these cases (Fig. 3). The lumbosacral integrity should be evaluated for the possibility of temporary vertical fixation to preserve lumbar motion, as we discussed in AO type C1 sacral fractures. In some cases, horizontal fixation alone is enough if secure fixation can be achieved, but weight-bearing ambulation might be delayed. For example, a 32-year-old female (case No. 15) had an AO type C2 fracture with an intact lumbosacral joint. She also had undergone plating for a right femoral shaft fracture, which precluded her from early weight bearing. So, this patient wound not get the full benefit of early weight bearing from lumbopelvic fixation. Then secure horizontal fixation was applied alone to permit ambulation with a wheelchair 1 month postoperatively, with progressive weight-bearing later.

**Fig. 4:**
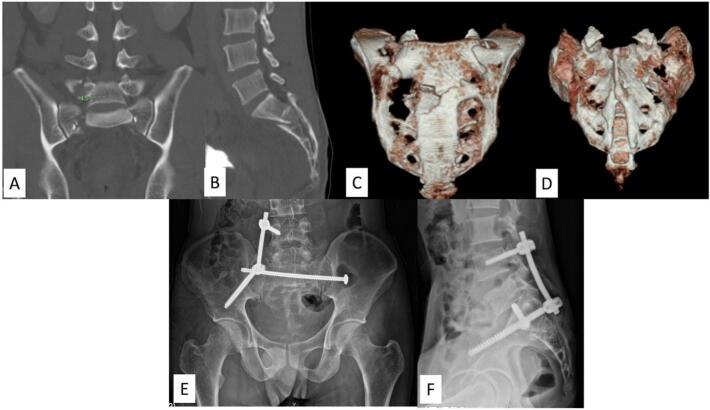
Demonstrating an AO type C3 sacral fracture with intact L5S1 facet joints (case No. 17). (A-D) Preoperative CT scan and CT reconstruction images showing a U-type fracture with a comminution fracture of the right sacral ala and right L5 pedicle. (E, F) Postoperative radiographs after transiliac-transsacral screw fixation with right triangular fixation.

**Fig. 5:**
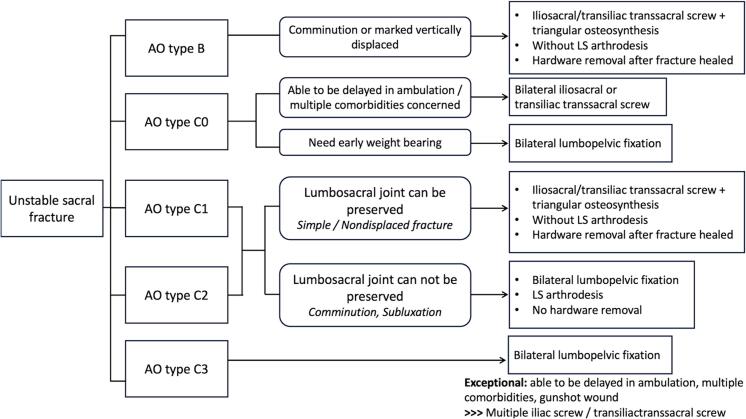
Surgical treatment guideline for an unstable sacral fracture.

An AO type C3 displaced U-type fracture with spinopelvic dissociation is often associated with high-energy mechanism injuries. Therefore, bilateral lumbopelvic fixation is commonly performed. Notwithstanding, in a carefully selected case, lumbosacral motion could be preserved. As shown in example case No.17 (Fig. 4), a male patient had an AO type C3 sacral fracture with comminution of the right sacral ala. The L5S1 facet joints were intact. Also, he had a calcaneal fracture, which impeded early ambulation. However, the patient was a young person who could tolerate some delay in ambulation. And secure fixation of the U-shape sacral fracture could be achieved. So, we applied a transiliac-transsacral screw for horizontal fixation and temporary right triangular osteosynthesis without arthrodesis. We planned for hardware removal later to preserve lumbosacral motion. Weight-bearing ambulation was delayed while waiting for the calcaneal fracture to heal. The summary of our surgical management strategy is shown in Fig. 5.

There could be some potential limitations to this work that arise from a retrospective design, i.e., there could have been a selection bias concerning the enrolled patients. The surgical methods were decided by the attending surgeon and were not controlled. Because our institute is a referral center, in some cases, we sent the patients back to nearby hospitals for their follow-ups after surgical fixation. We tried to contact those hospitals to get clinical information and functional scores, but some data were still missing. Also, in our institute, we do not perform imaging such as a CT scan in every case to confirm radiographic union. We defined successful treatment after fixation using ODI functional scores, NRS pain scores, and plain radiographic evidence that showed no loosening or need for reoperation. Further prospective studies comparing the surgical method for each specific fracture will shed light on the appropriate surgical strategy.

## 5 Conclusion

Unstable sacral fractures with posterior pelvic and spinopelvic instability are rare conditions that usually result from high-energy injuries. The actual knowledge and treatment guidelines are still controversial at some points due to the rarity of this injury. We have proposed the surgical management strategy for unstable sacral fractures based on three factors: 1) fracture morphology, 2) lumbosacral integrity, and 3) concomitant injury.

## Consent

Written informed consent was obtained from the patient for publication of this case report and accompanying images. A copy of the written consent is available for review by the Editor-in-Chief of this journal on request.

## Ethical approval

This study was approved by the Ethics Committee and Institutional Review Board (REC.65-411-11-1) of Prince of Songkla University Hospital, Hatyai, Thailand on 31 December 2022.

## Funding

No funds were received in support of this work.

## Author contribution

Chonnipa Siri-archawawat: data collection, analysis, and first draft of the manuscript.

Weera Chaiyamongkol: study conception and design, material preparation, analysis, and final editing of the manuscript.

## Guarantor

Weera Chaiyamongkol.

## Research registration number

REC.65-411-11-1.

## Conflict of interest statement

No benefits in any form have been or will be received from a commercial party related directly or indirectly to the subject of this manuscript.

## Data Availability

The data generated during the study period is provided in the manuscript. Additional data are available upon request.
